# Supplementary material for: Ribosome impairment regulates intestinal stem cell identity via ZAKɑ activation
Source: Nat Commun. 2022 Aug 2;13:4492. doi: 10.1038/s41467-022-32220-4 (PMC9345940; doi:10.1038/s41467-022-32220-4)
Supplement: Supplementary file 1 — Supplementary Information [file 41467_2022_32220_MOESM1_ESM.pdf]

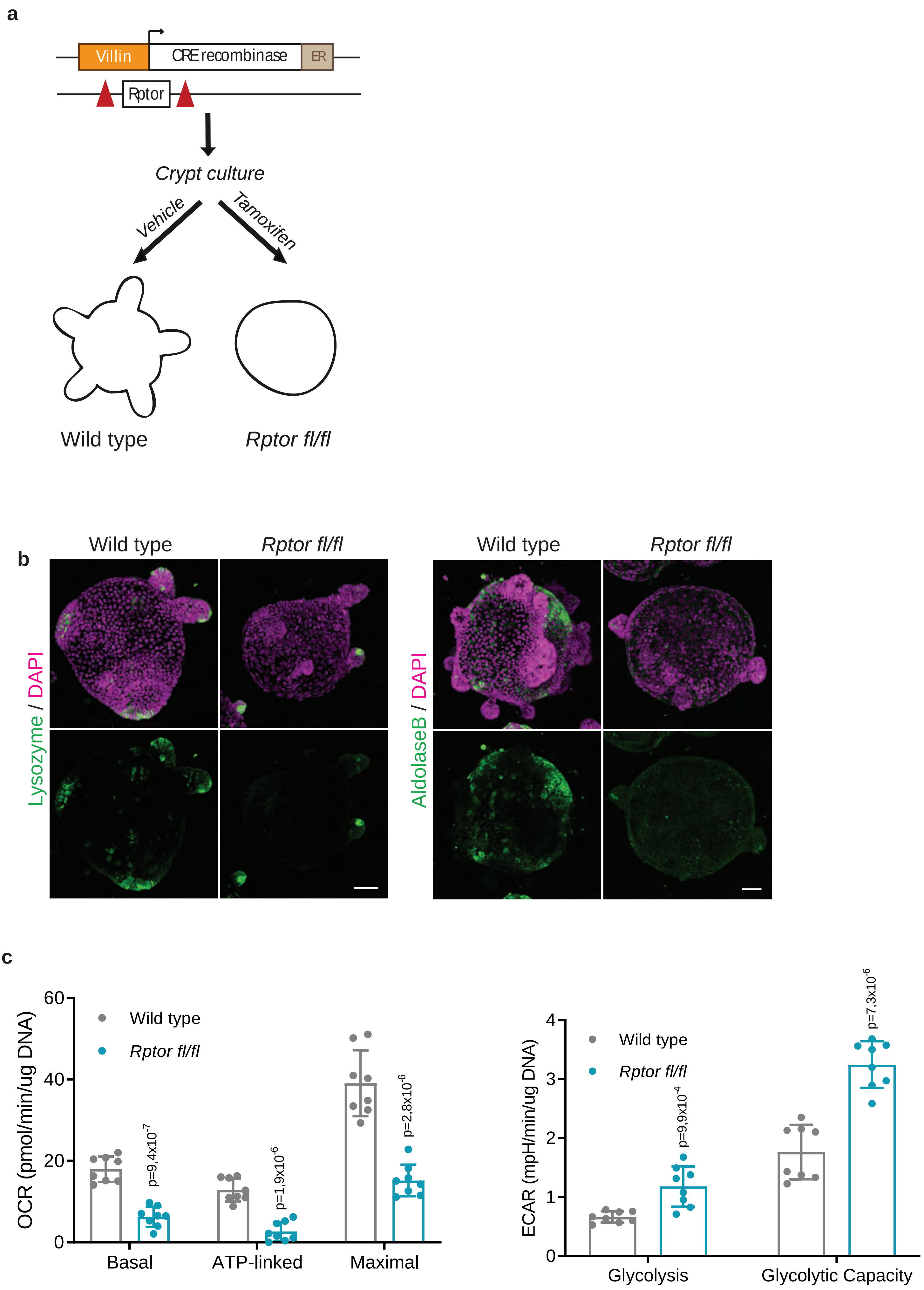
a
Villin
CRE recombinase
ER
Rptor
Crypt culture
Vehicle
Tamoxifen
Wild type
Rptor fl/fl
b
Wild type
Rptor fl/fl
Wild type
Rptor fl/fl
Lysozyme / DAPI
AldolaseB / DAPI
c
OCR (pmol/min/ug DNA)
Wild type
Rptor fl/fl
p=9,4x10-7
p=1,9x10-6
p=2,8x10-6
Basal
ATP-linked
Maximal
ECAR (mpH/min/ug DNA)
p=9,9x10-4
p=7,3x10-6
Glycolysis
Glycolytic Capacity

Supplementary Figure 1 - Experimental workflow, immunostainings and seahorse analysis of crypt cultures from wild type and Rptor$^{fl/fl}$ mice - Related to Figure 1

A. Experimental workflow of the generation of crypt cultures from VillinCre^{ERT2}Rptor$^{fl/fl}$ mice, showing morphological differences caused by Rptor deletion.

B. Representative 3D-reconstructed confocal images of wild type and Rptor$^{fl/fl}$ organoids show significant reduction of differentiation markers Lysozyme (left panel, green) and Aldolase (right panel, green). Dapi is used to visualize the nuclei (magenta). Scale bar is 50um.

C. OCR and ECAR analyses reveal decreased respiration and increased glycolysis in Rptor$^{fl/fl}$ organoids compared to WT. Mean and standard deviation are shown (n = 2 biological replicates each accessed in technical quadriplicates). p values were determined using a two-tailed t-test. Related to Figure 1F.

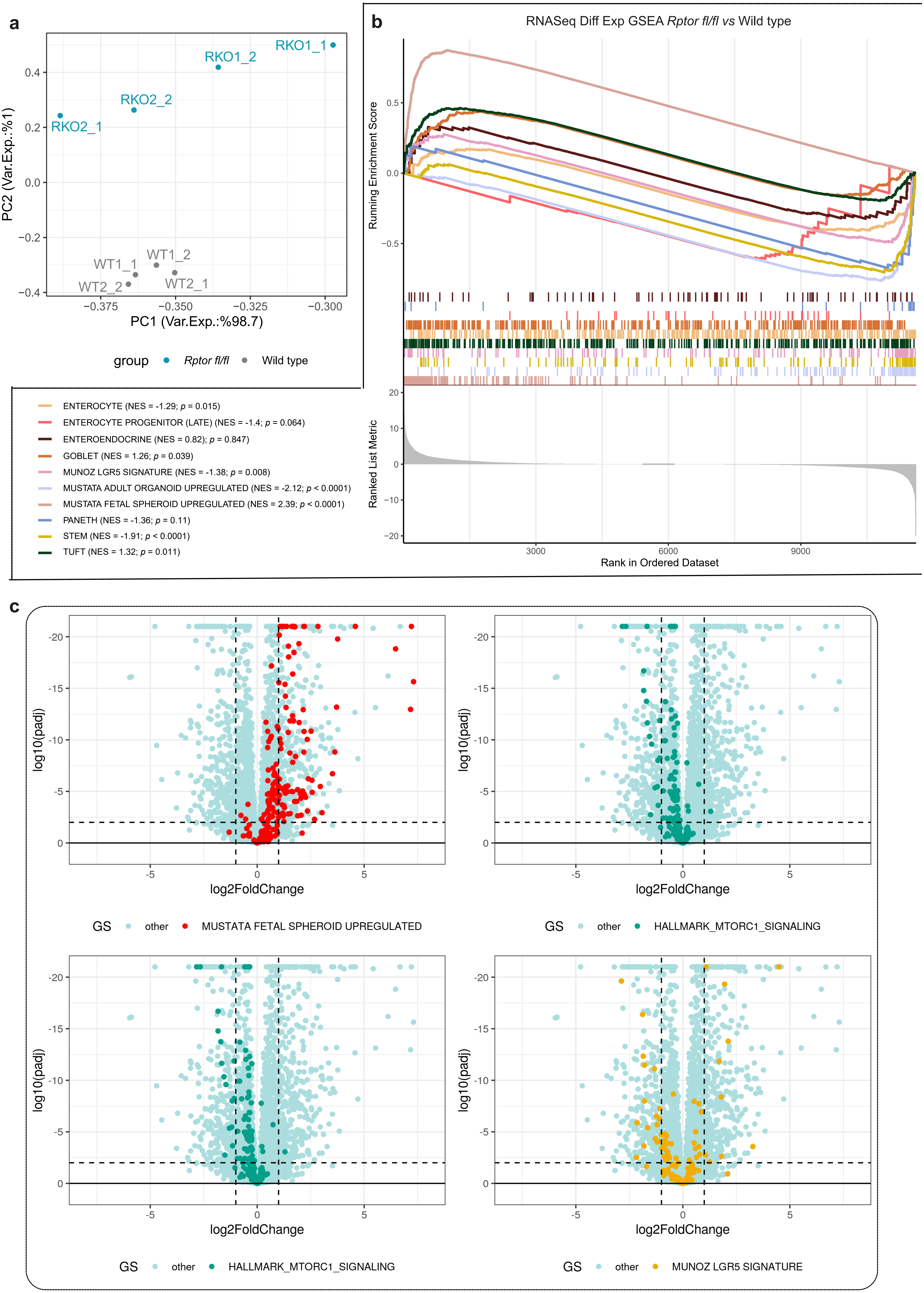
a
RKO1_1
RKO1_2
RKO2_2
RKO2_1
WT1_2
WT1_1
WT2_2
WT2_1
PC2 (Var.Exp.:%1)
PC1 (Var.Exp.:%98.7)
group Rptor fl/fl Wild type
b
RNASeq Diff Exp GSEA Rptor fl/fl vs Wild type
Running Enrichment Score
Ranked List Metric
Rank in Ordered Dataset
ENTEROCYTE (NES = -1.29; p = 0.015)
ENTEROCYTE PROGENITOR (LATE) (NES = -1.4; p = 0.064)
ENTEROENDOCRINE (NES = 0.82); p = 0.847)
GOBLET (NES = 1.26; p = 0.039)
MUNOZ LGR5 SIGNATURE (NES = -1.38; p = 0.008)
MUSTATA ADULT ORGANOID UPREGULATED (NES = -2.12; p < 0.0001)
MUSTATA FETAL SPHEROID UPREGULATED (NES = 2.39; p < 0.0001)
PANETH (NES = -1.36; p = 0.11)
STEM (NES = -1.91; p < 0.0001)
TUFT (NES = 1.32; p = 0.011)
c
log10(padj)
log2FoldChange
GS other MUSTATA FETAL SPHEROID UPREGULATED
GS other HALLMARK_MTORC1_SIGNALING
GS other HALLMARK_MTORC1_SIGNALING
GS other MUNOZ LGR5 SIGNATURE

Supplementary Figure 2 - Summary of RNAseq analysis of Rptor$^{fl/fl}$ and wild type organoids - Related to Figure 1

A. PCA plot for RNAseq data performed in Rptor$^{fl/fl}$ and WT organoids, 2 technical replicates from 2 biological replicates for each condition.

B. Gene Set Enrichment Analysis based on RNASeq differential expression data comparing Rptor$^{fl/fl}$ organoids to WT (n=4 from 2 biological replicates for each). Enrichment is shown for transcriptional signatures related to stemness, fetal signature and various differentiated types of cells. p values were determined using the *DESEQ2* package.

C. Differential expression results of Rptor$^{fl/fl}$ and WT comparison, presented as volcano plots where genesets from Fig. 1G are highlighted in separate panels. p values were determined using the *DESEQ2* package.

a

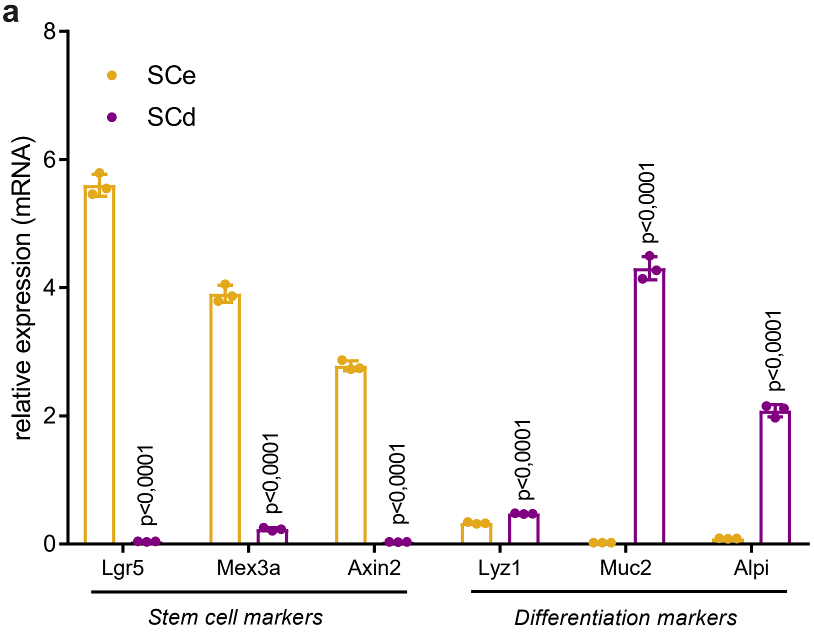

SCe
SCd
relative expression (mRNA)
0
2
4
6
8
p<0,0001
p<0,0001
p<0,0001
p<0,0001
p<0,0001
p<0,0001
Lgr5
Mex3a
Axin2
Lyz1
Muc2
Alpi
Stem cell markers
Differentiation markers

b

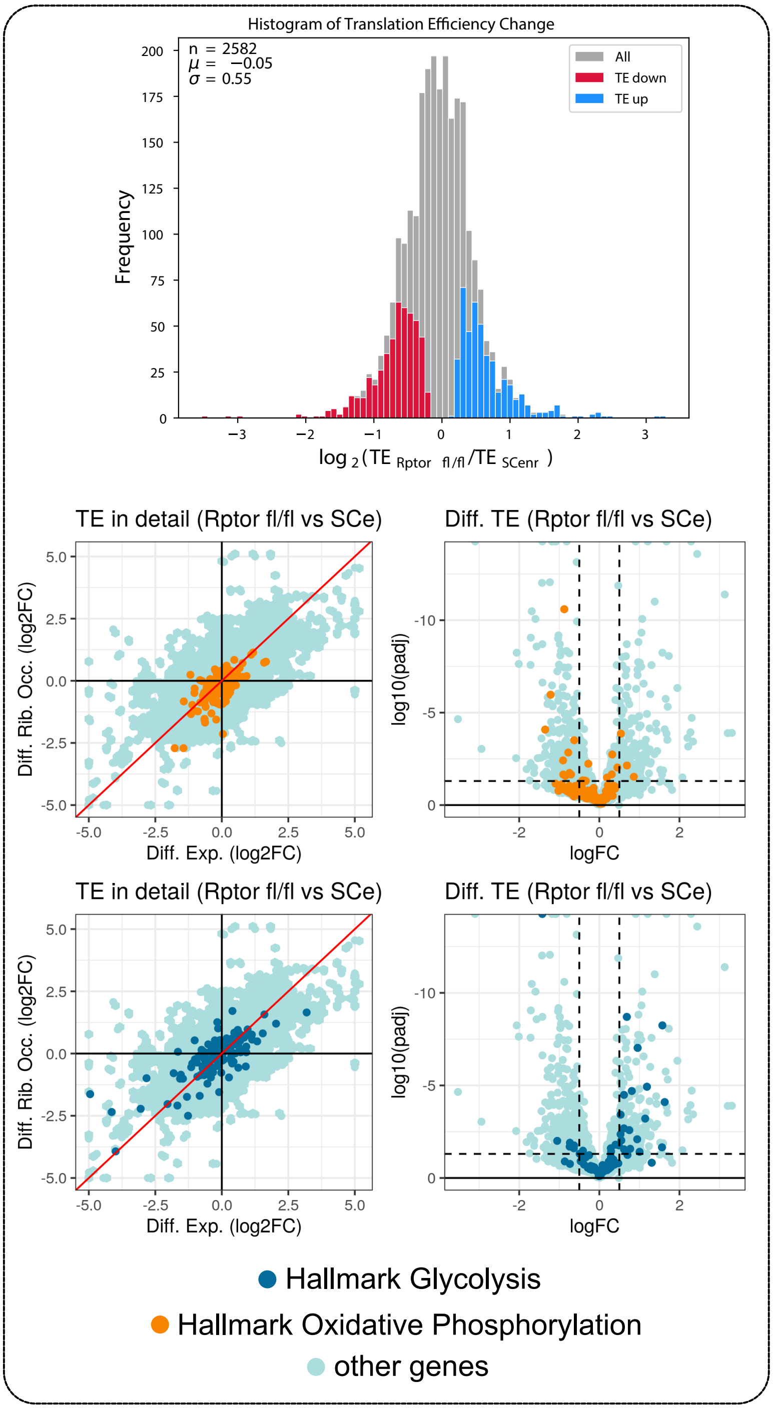

Histogram of Translation Efficiency Change
n = 2582
μ = −0.05
σ = 0.55
All
TE down
TE up
Frequency
log 2 (TE Rptor fl/fl /TE SCenr)
TE in detail (Rptor fl/fl vs SCe)
Diff. Rib. Occ. (log2FC)
Diff. Exp. (log2FC)
Diff. TE (Rptor fl/fl vs SCe)
log10(padj)
logFC
TE in detail (Rptor fl/fl vs SCe)
Diff. Rib. Occ. (log2FC)
Diff. Exp. (log2FC)
Diff. TE (Rptor fl/fl vs SCe)
log10(padj)
logFC
Hallmark Glycolysis
Hallmark Oxidative Phosphorylation
other genes

Supplementary Figure 3 - Summary of translation efficiency analysis of Rptor$^{fl/fl}$ and SC enriched organoids - Related to Figure 2

A. RT-qPCR analysis of individual genes related to stem (Lgr5, Mex3a and Axin2) and differentiation (Lyz1, Muc2 and Alpi) of SCe and SCd organoids normalized to WT, using Hprt as a reference. Mean and standard error of the mean are shown (n = 3 biological replicates each accessed in technical triplicates).p values were determined using a two-tailed t-test.

B. Summary of the differential translation efficiency analysis for Rptor$^{fl/fl}$ vs SCe comparison. Top row panel shows the histogram of translation efficiency changes across conditions where significant genes are highlighted. Other rows present the details of the differential TE analysis highlighting the directionality of specific genesets from Fig2c (one geneset per row). In these rows, the left panel compares the gene-specific expression and ribosome occupation changes whereas the right panel summarizes the statistics of differential translation efficiency analysis.

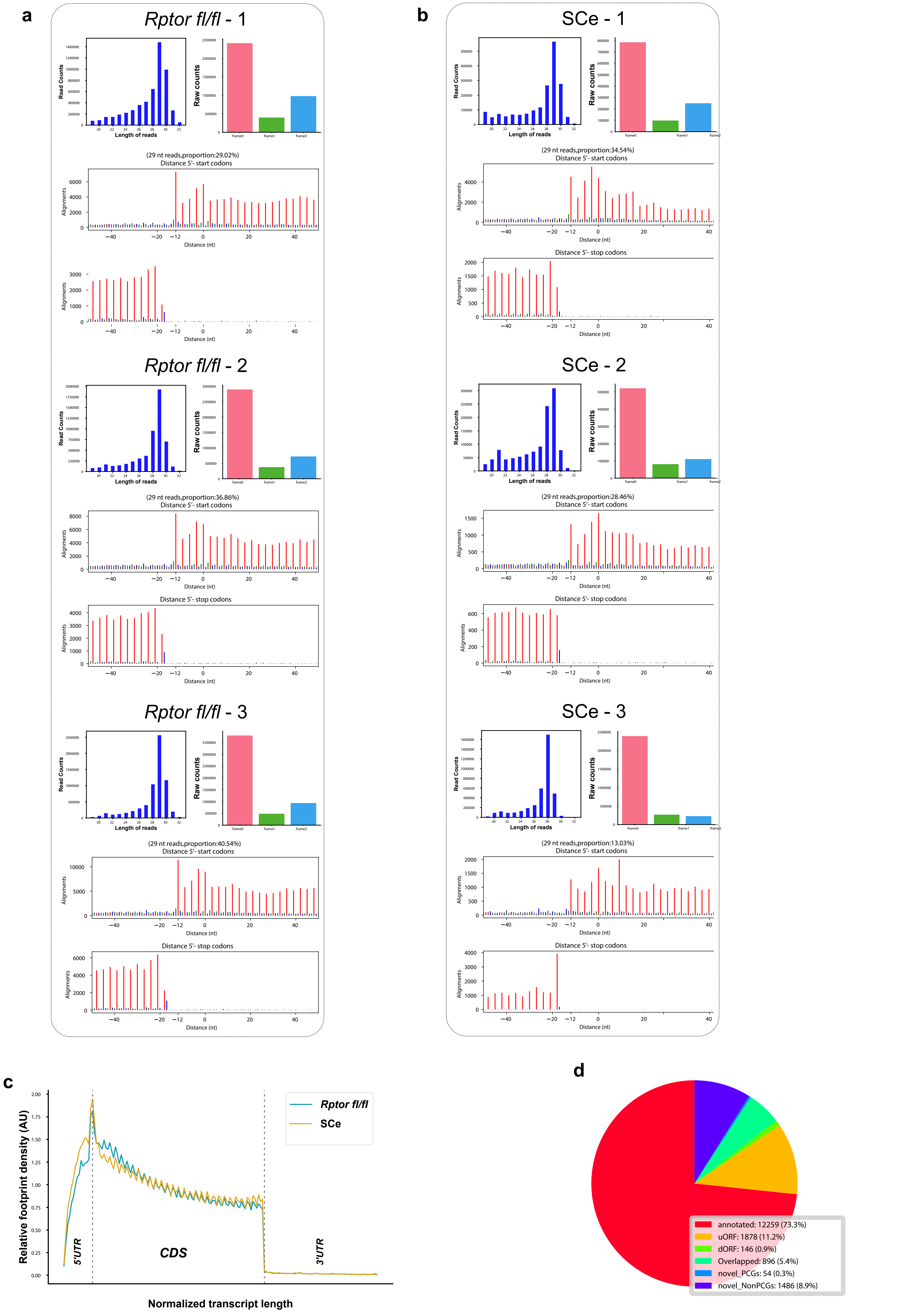
a
Rptor fl/fl - 1
Read Counts
Length of reads
Raw counts
frame0
frame1
frame2
(29 nt reads,proportion:29.02%)
Distance 5'- start codons
Alignments
Distance (nt)
Rptor fl/fl - 2
(29 nt reads,proportion:36.86%)
Distance 5'- start codons
Distance 5'- stop codons
Rptor fl/fl - 3
(29 nt reads,proportion:40.54%)
Distance 5'- start codons
Distance 5'- stop codons
b
SCe - 1
(29 nt reads,proportion:34.54%)
Distance 5'- start codons
Distance 5'- stop codons
SCe - 2
(29 nt reads,proportion:28.46%)
Distance 5'- start codons
Distance 5'- stop codons
SCe - 3
(29 nt reads,proportion:13.03%)
Distance 5'- start codons
Distance 5'- stop codons
c
Relative footprint density (AU)
Rptor fl/fl
SCe
5'UTR
CDS
3'UTR
Normalized transcript length
d
annotated: 12259 (73.3%)
uORF: 1878 (11.2%)
dORF: 146 (0.9%)
Overlapped: 896 (5.4%)
novel_PCGs: 54 (0.3%)
novel_NonPCGs: 1486 (8.9%)

Supplementary Figure 4 - Quality Control plots for Ribo-seq experiments performed in Rptor$^{fl/fl}$ and SC enriched organoids - Related to Figure 2

A. Quality Control plots for Ribo-seq experiments performed in Rptor$^{fl/fl}$ organoid samples. QC plots, generated by the RiboCode tool [77], include read length histograms, in & out of frame statistics and periodicity-plots of mRNA-mapped reads separately for each sample.

B. Ribo-seq QC plots for SC enriched organoids.

C. Positional metaplot showing the relative position of mRNA mapped-reads, averaged over 3 samples of each group.

D. RiboCode ORF prediction statistics using the Ribo-seq data from all 9 samples. Raw numbers and percentages of different classes of ORFs are presented in a pie chart.

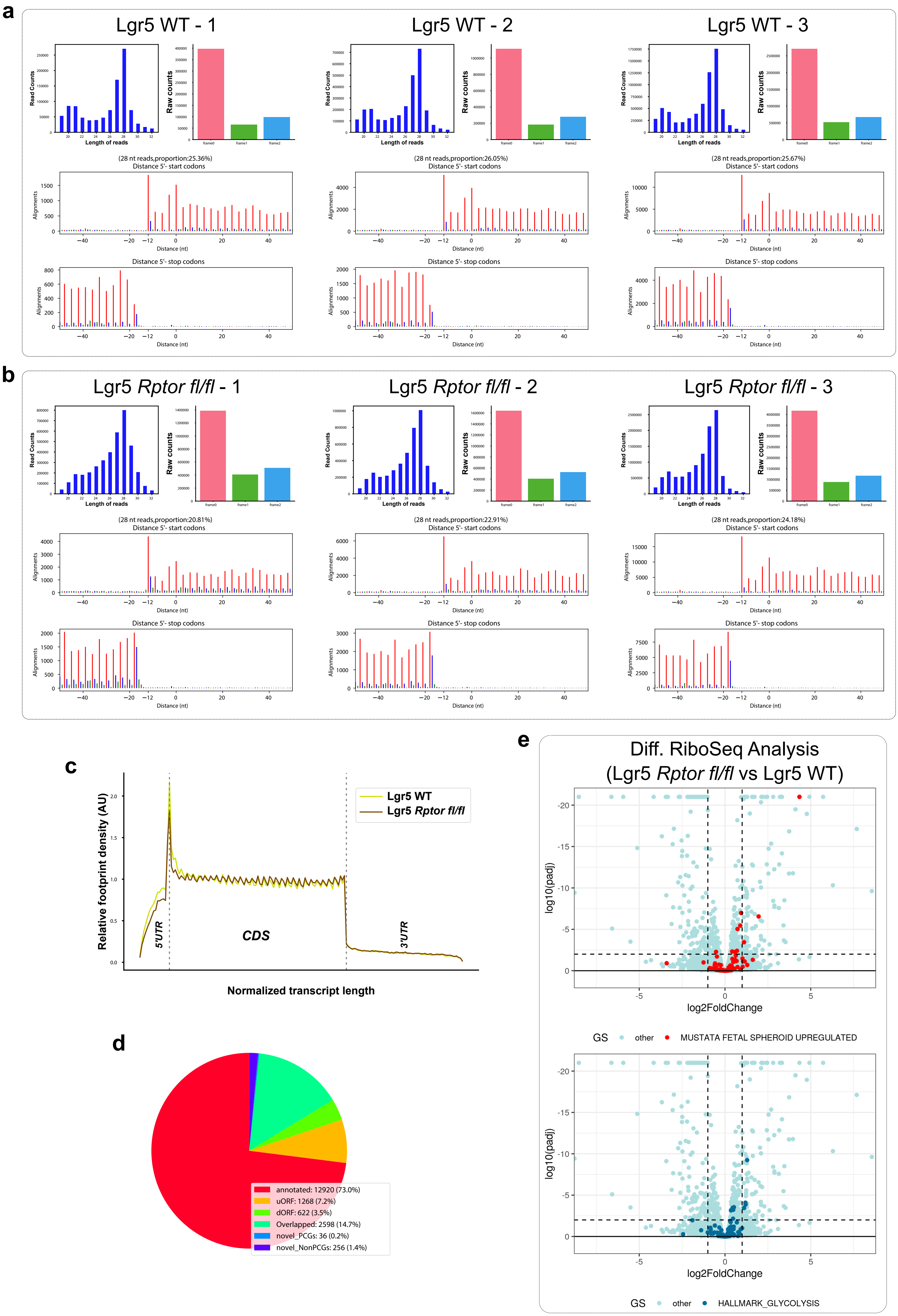
a
Lgr5 WT - 1
Lgr5 WT - 2
Lgr5 WT - 3
Read Counts
Length of reads
Raw counts
frame0
frame1
frame2
(28 nt reads,proportion:25.36%)
(28 nt reads,proportion:26.05%)
(28 nt reads,proportion:25.67%)
Distance 5'- start codons
Distance 5'- stop codons
Alignments
Distance (nt)
b
Lgr5 *Rptor fl/fl* - 1
Lgr5 *Rptor fl/fl* - 2
Lgr5 *Rptor fl/fl* - 3
(28 nt reads,proportion:20.81%)
(28 nt reads,proportion:22.91%)
(28 nt reads,proportion:24.18%)
c
Relative footprint density (AU)
Lgr5 WT
Lgr5 *Rptor fl/fl*
5'UTR
CDS
3'UTR
Normalized transcript length
d
annotated: 12920 (73.0%)
uORF: 1268 (7.2%)
dORF: 622 (3.5%)
Overlapped: 2598 (14.7%)
novel_PCGs: 36 (0.2%)
novel_NonPCGs: 256 (1.4%)
e
Diff. RiboSeq Analysis
(Lgr5 *Rptor fl/fl* vs Lgr5 WT)
log10(padj)
log2FoldChange
GS other MUSTATA FETAL SPHEROID UPREGULATED
GS other HALLMARK_GLYCOLYSIS

Supplementary Figure 5 - Quality Control plots for Ribo-seq experiments performed in Lgr5Cre^{ERT2};Rptor$^{fl/fl}$;RiboTag$^{HA/HA}$ animals - Related to Figure 2

A. Quality Control plots for in vivo Ribo-seq experiments performed in Lgr5 WT intestine samples. QC plots include read length histograms, in & out of frame statistics and periodicity-plots of mRNA-mapped reads separately for each sample.

B. Ribo-seq QC plots for Lgr5Cre^{ERT2}Rptor$^{fl/fl}$ intestine samples.

C. Positional metaplot showing the relative position of mRNA mapped-reads, averaged over 3 samples of each group.

D. RiboCode ORF prediction statistics using the Ribo-seq data from all 6 in vivo samples. Raw numbers and percentages of different classes of ORFs are presented in a pie chart.

E. Differential ribosomal occupation results for Lgr5Cre^{ERT2}Rptor$^{fl/fl}$ and Lgr5Cre^{ERT2}Rptor$^{+/+}$ comparison, presented as volcano plots where genesets from Fig.4f are highlighted in separate panels. p values were determined using the *DESEQ2* package.

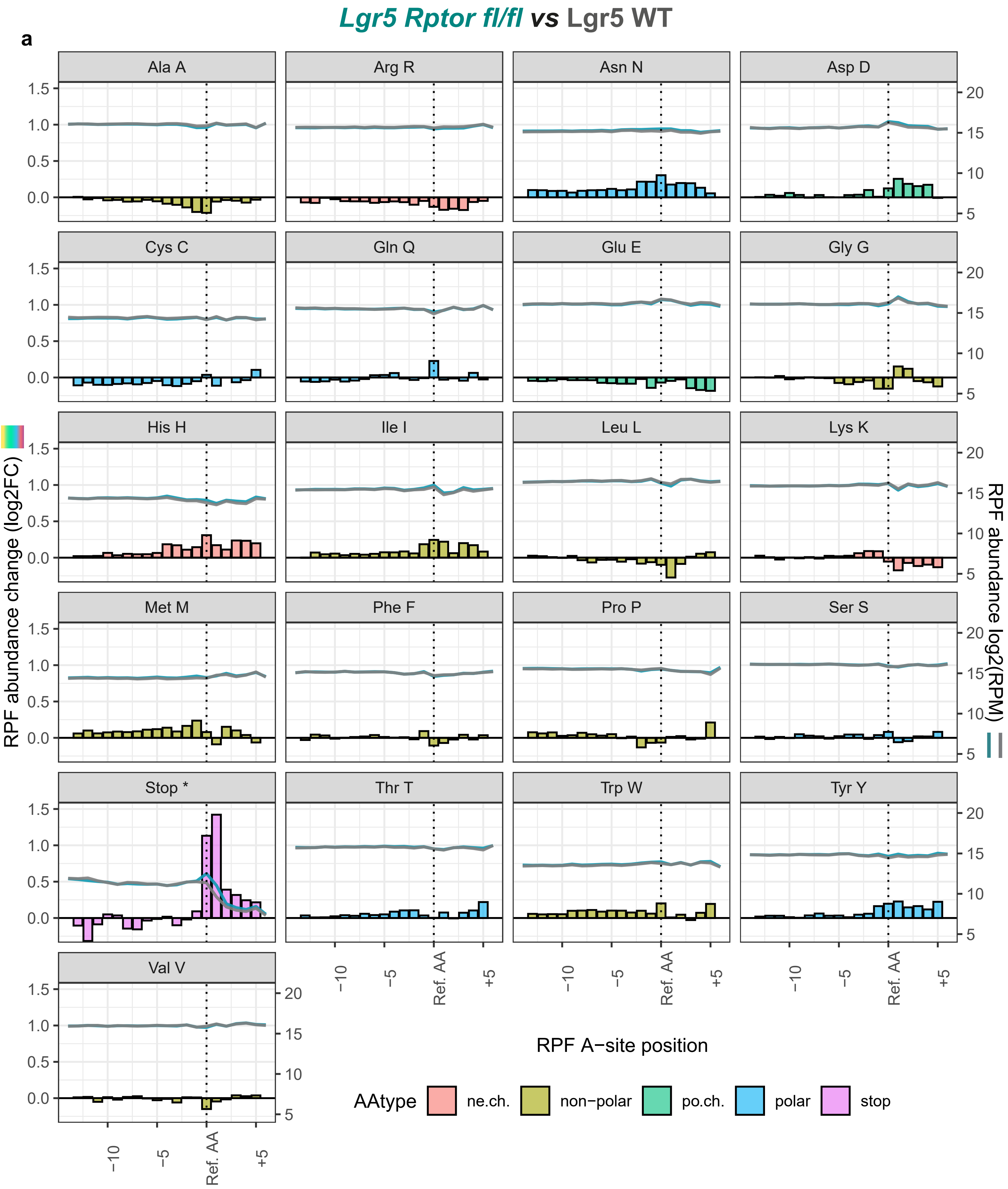

Lgr5 Rptor fl/fl vs Lgr5 WT
a
Ala A
Arg R
Asn N
Asp D
Cys C
Gln Q
Glu E
Gly G
His H
Ile I
Leu L
Lys K
Met M
Phe F
Pro P
Ser S
Stop *
Thr T
Trp W
Tyr Y
Val V
RPF abundance change (log2FC)
RPF abundance log2(RPM)
RPF A−site position
−10
−5
Ref. AA
+5
AAtype
ne.ch.
non−polar
po.ch.
polar
stop

Supplementary Figure 6 – Codon-specific analysis of RPF distribution upon Rptor deletion - Related to Figure 2

A. Distribution of RPFs along transcripts in various codons in the Lgr5Cre^{ERT2}Rptor$^{fl/fl}$RPL22.HA mice (blue) compared with the Lgr5Cre^{ERT2}RPL22.HA (gray). Barplot depicts RPF abundance change (log2FC) between the two conditions and lines show total RPF abundance (%), for which RPFs are grouped based on their A-site position.

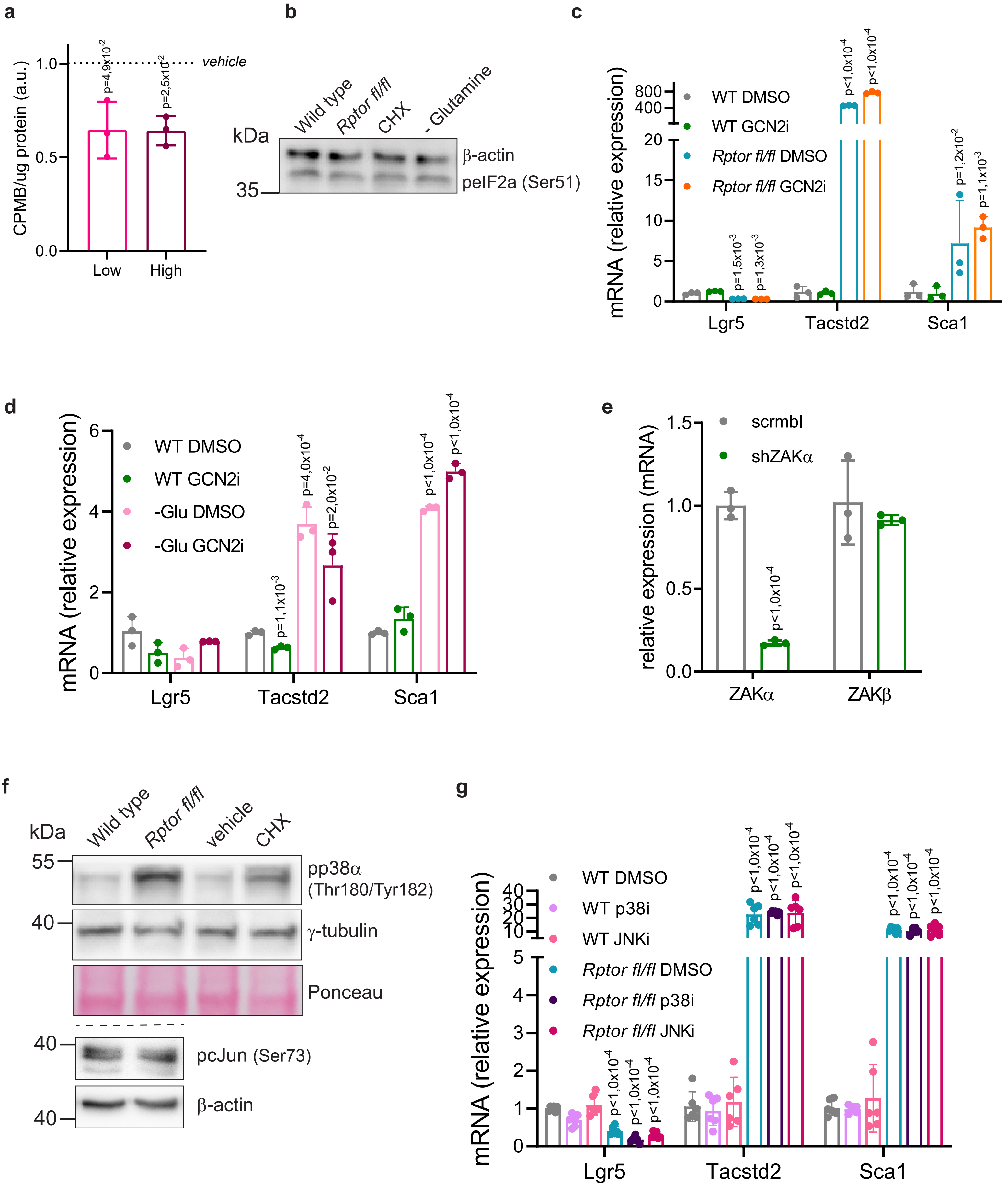
a
b
c
CPMB/ug protein (a.u.)
vehicle
p=4,9x10^-2
p=2,5x10^-2
Low
High
kDa
Wild type
Rptor fl/fl
CHX
- Glutamine
β-actin
peIF2a (Ser51)
35
mRNA (relative expression)
WT DMSO
WT GCN2i
Rptor fl/fl DMSO
Rptor fl/fl GCN2i
p=1,5x10^-3
p=1,3x10^-3
p<1,0x10^-4
p=1,2x10^-2
p=1,1x10^-3
Lgr5
Tacstd2
Sca1
d
-Glu DMSO
-Glu GCN2i
p=1,1x10^-3
p=4,0x10^-4
p=2,0x10^-2
e
scrmbl
shZAKα
relative expression (mRNA)
ZAKα
ZAKβ
f
vehicle
pp38α (Thr180/Tyr182)
γ-tubulin
Ponceau
pcJun (Ser73)
g
WT p38i
WT JNKi
Rptor fl/fl p38i
Rptor fl/fl JNKi

Supplementary Figure 7 – Cell identity switch caused by Rptor deletion is not mediated by the canonical eIF2a or GCN2 pathways - Related to Figures 3

A. Incorporation of ^{35}S-methionine shows decreased protein synthesis in WT organoids treated with low (0,015ug/ml) and high (100ug/ml) of cycloheximide. Mean and standard deviation are shown (n = 1 biological replicate accessed in technical triplicates). * p values were determined using a two-tailed t-test.

B. Western blot analysis shows that Raptor deletion, cycloheximide treatment (0,015ug/ml) or glutamine deprivation do not significantly affect peIF2α (Ser51), compared to WT. β-actin serves as loading control. Blots were done on one animal.

C. RT-qPCR analysis of individual genes related to stem (Lgr5) and fetal-like state (Tacstd2 and Sca1) of WT and Rptor$^{fl/fl}$ organoids, treated with GCN2 inhibitor (A-92, 2uM, 24h)). Hprt is used as a reference. Mean and standard deviation are shown (n = 1 biological replicate accessed in technical triplicates). p values were determined using a two-tailed t-test.

D. RT-qPCR analysis of individual genes related to stem (Lgr5) and fetal-like state (Tacstd2 and Sca1) of WT organoids and organoids grown without glutamine, treated with GCN2 inhibitor (A-92, 2uM, 24h)). Hprt is used as a reference. Mean and standard deviation are shown (n = 1 biological replicate accessed in technical triplicates). p values were determined using a two-tailed t-test.

E. qRT-PCR confirmed the reduction of ZAKα mRNA following expression of stable shRNAs against ZAKα. No significant differences are seen in ZAKβ levels. Mean and standard error of the mean are shown (n = 1 biological replicate accessed in technical triplicates). p values were determined using a two-tailed t-test.

F. Western blot analysis showing activation of p38α in Rptor$^{fl/fl}$ and low dose cycloheximide treated organoids compared to WT. γ-tubulin and Ponceau serve as loading control. Bottom panel shows no differences in cJun activation upon Rptor deletion compared with WT. β-actin serves as loading control. Blots were done on one animal.

G. RT-qPCR analysis of individual genes related to stem (Lgr5) and fetal-like state (Tacstd2 and Sca1) of WT organoids treated with inhibitors for p38 (SB203580, 10uM, 24h) and JNK (JNK-IN-8, 1uM for 24h). Hprt is used as a reference. Mean and standard error of the mean are shown (n = 2 biological replicates accessed in technical triplicates). p values were determined using a two-tailed t-test.

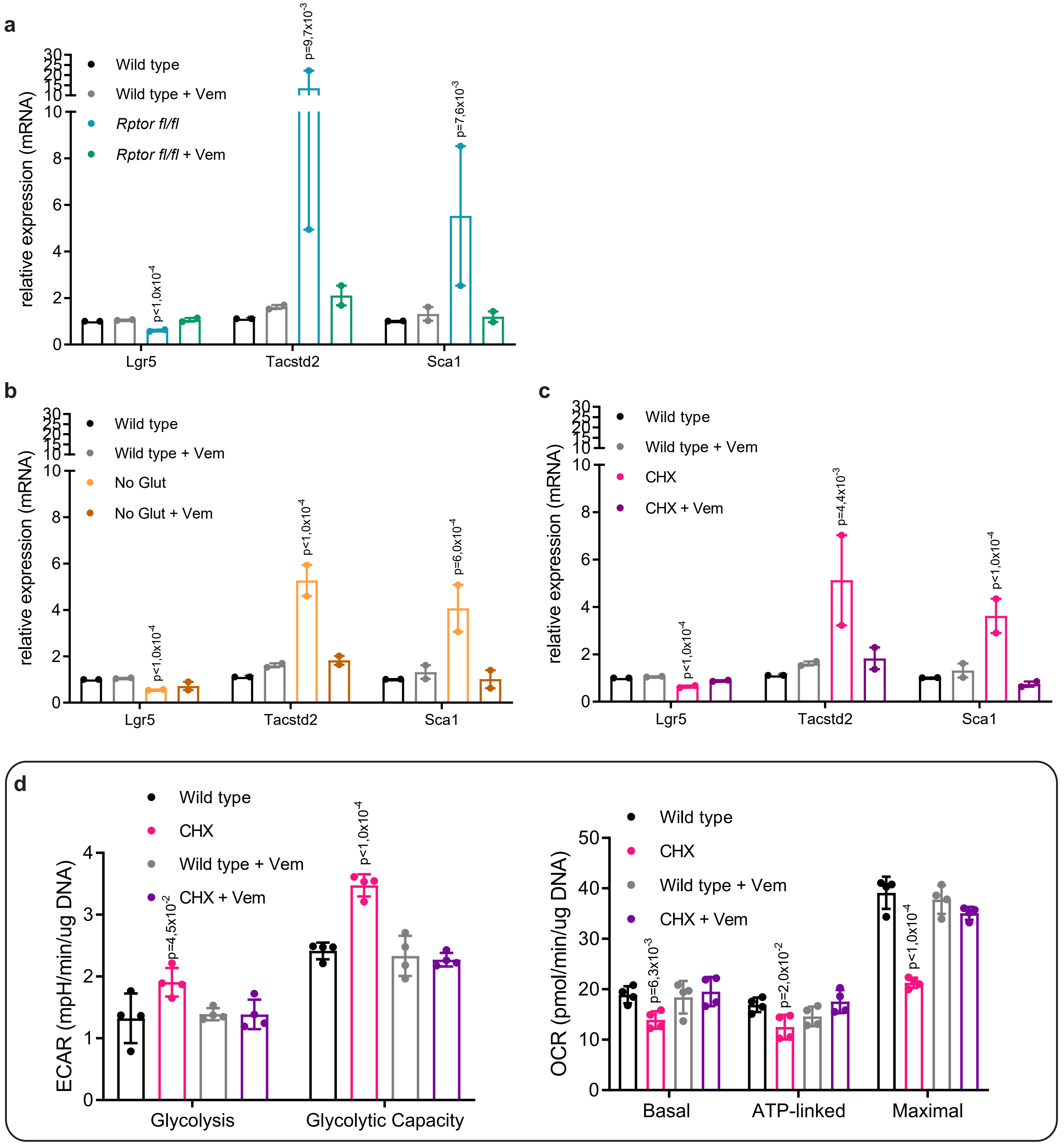

a
Wild type
Wild type + Vem
Rptor fl/fl
Rptor fl/fl + Vem
relative expression (mRNA)
p<1,0x10-4
p=9,7x10-3
p=7,6x10-3
Lgr5
Tacstd2
Sca1
b
Wild type
Wild type + Vem
No Glut
No Glut + Vem
relative expression (mRNA)
p<1,0x10-4
p<1,0x10-4
p=6,0x10-4
Lgr5
Tacstd2
Sca1
c
Wild type
Wild type + Vem
CHX
CHX + Vem
relative expression (mRNA)
p<1,0x10-4
p=4,4x10-3
p<1,0x10-4
Lgr5
Tacstd2
Sca1
d
Wild type
CHX
Wild type + Vem
CHX + Vem
ECAR (mpH/min/ug DNA)
p=4,5x10-2
p<1,0x10-4
Glycolysis
Glycolytic Capacity
OCR (pmol/min/ug DNA)
p=6,3x10-3
p=2,0x10-2
p<1,0x10-4
Basal
ATP-linked
Maximal

Supplementary Figure 8 - ZAKα-mediated ribosome impairment affect intestinal stem cell identity and metabolism - Related to Figure 3

A. RT-qPCR analysis shows that vemurafenib treatment (1uM for 1 hour) is enough to rescue the decrease of Lgr5 and increase in Tacstd2 and Sca1 seen in $Rptor^{fl/fl}$ organoids. Hprt is used as a reference. Mean and standard error of the mean are shown (n = 2 biological replicates each accessed in technical triplicates). p values were determined using a two-tailed t-test.

B. RT-qPCR analysis shows that vemurafenib treatment (1uM for 1 hour) is enough to rescue the decrease of Lgr5 and increase in Tacstd2 and Sca1 seen in organoids cultured in glutamine-free media. Hprt is used as a reference. Mean and standard error of the mean are shown (n = 2 biological replicates each accessed in technical triplicates). p values were determined using a two-tailed t-test.

C. RT-qPCR analysis shows that vemurafenib treatment (1uM for 1 hour) is enough to rescue the decrease of Lgr5 and increase in Tacstd2 and Sca1 seen in organoids treated with low dose cycloheximide (0,015ug/ml) for 30min . Hprt is used as a reference. Mean and standard error of the mean are shown (n = 2 biological replicates each accessed in technical triplicates). p values were determined using a two-tailed t-test.

D. ECAR and OCR analyses reveal that inhibiting ZAKα by treating organoids with vemurafenib (1uM for 1 hour) rescues the metabolic changes caused by low dose of cycloheximide (0,015ug/ml 30min). Mean and standard deviation are shown (n = 1 biological replicate accessed in technical quadriplicates). p values were determined using a two-tailed t-test.

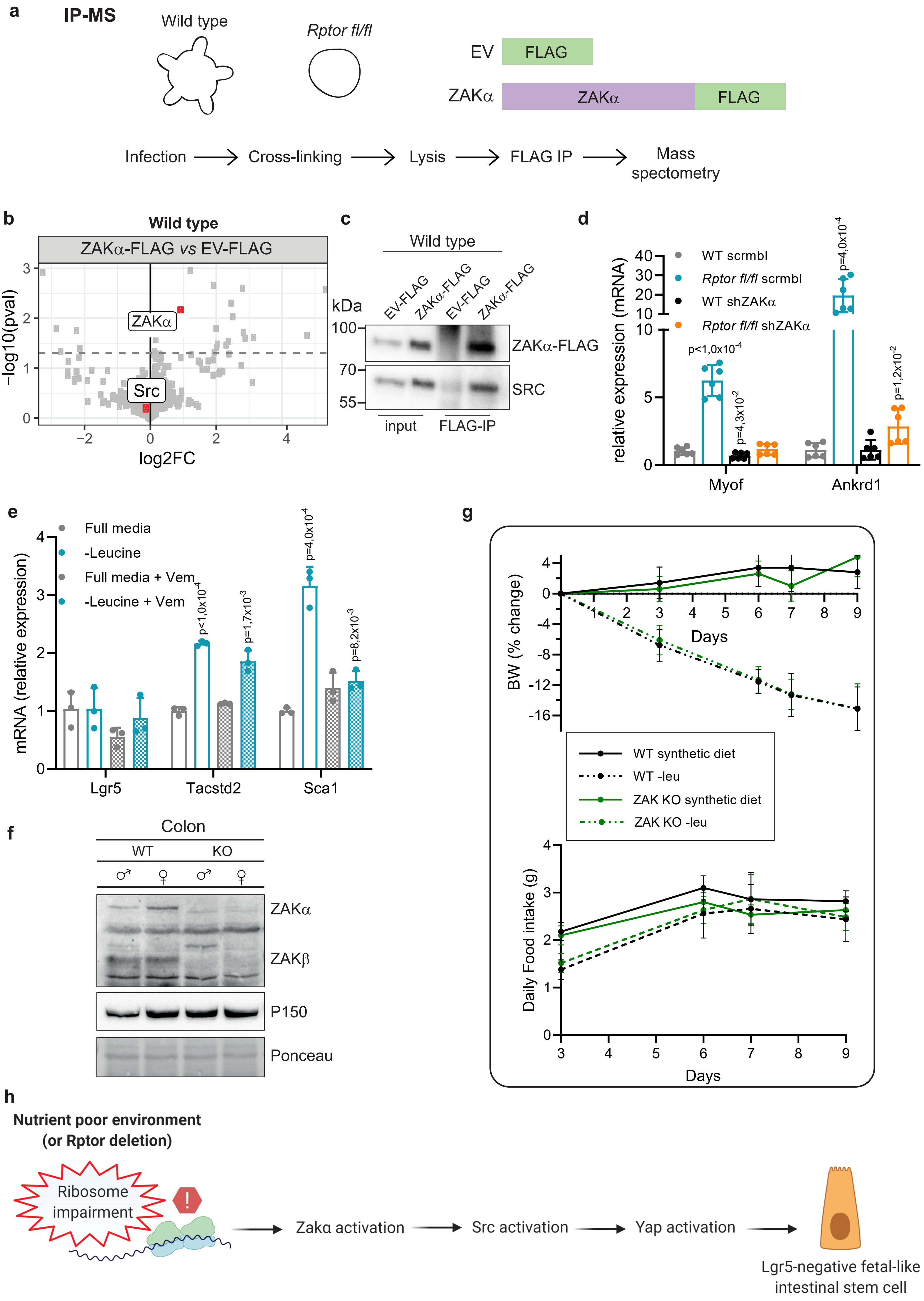
a
IP-MS
Wild type
Rptor fl/fl
EV
FLAG
ZAKα
ZAKα
FLAG
Infection
Cross-linking
Lysis
FLAG IP
Mass spectometry
b
Wild type
ZAKα-FLAG vs EV-FLAG
−log10(pval)
ZAKα
Src
log2FC
c
Wild type
kDa
EV-FLAG
ZAKα-FLAG
EV-FLAG
ZAKα-FLAG
100
70
55
ZAKα-FLAG
SRC
input
FLAG-IP
d
relative expression (mRNA)
WT scrmbl
Rptor fl/fl scrmbl
WT shZAKα
Rptor fl/fl shZAKα
p<1,0x10-4
p=4,3x10-2
p=4,0x10-4
p=1,2x10-2
Myof
Ankrd1
e
mRNA (relative expression)
Full media
-Leucine
Full media + Vem
-Leucine + Vem
p<1,0x10-4
p=1,7x10-3
p=4,0x10-4
p=8,2x10-3
Lgr5
Tacstd2
Sca1
f
Colon
WT
KO
♂
♀
♂
♀
ZAKα
ZAKβ
P150
Ponceau
g
BW (% change)
Days
WT synthetic diet
WT -leu
ZAK KO synthetic diet
ZAK KO -leu
Daily Food intake (g)
Days
h
Nutrient poor environment
(or Rptor deletion)
Ribosome impairment
Zakα activation
Src activation
Yap activation
Lgr5-negative fetal-like intestinal stem cell

Supplementary Figure 9 - ZAKα activates the Src-Yap axis during ribosome impairment and results in ISCs identity switch *in vivo* during leucine deprivation - Related to Figure 4

A. Experimental workflow of the affinity purification strategy (IP-MS) for FLAG tagged ZAKα in wild type and $Rptor^{fl/fl}$ organoids. Replicates of the affinity purification were digested with trypsin and subjected to LC-MS/MS to quantitatively identify specific ZAKα interactor. A FLAG tagged empty vector is used as a control.

B. Volcano plot depicting differentially enriched interactors of ZAKα in wild type organoids, using RIME-MS (n = 2 biological replicates). A FLAG tagged empty vector bait is used as a control. Significance when p-value ⩽ 0,05 (t-test, two-tailed).

C. Immunoprecipitation of ZAKα-FLAG shows residual interaction with Src in wild type organoids. A FLAG tagged empty vector is used as a control. Experiment was done on one animal.

D. RT-qPCR analysis shows activation of Yap-target genes (Myof and Ankrd1) in $Rptor^{fl/fl}$ organoids compared to wild type. Hprt is used as a reference. Mean and standard error of the mean are shown (n = 2 biological replicates each accessed in technical triplicates). p values were determined using a two-tailed t-test.

E. RT-qPCR analysis shows activation of fetal markers Tacstd2 and Sca1 in human colon organoids cultured in leucine-free media. Treatment with vemurafenib (1uM for 1 hour) partially rescues this activation. Hprt is used as a reference. Mean and standard error of the mean are shown (n = biological replicate accessed in technical triplicates). p values were determined using a two-tailed t-test.

F. Western blot analysis confirming deletion of ZAKα *in vivo*. Ponceau and P150 serve as loading control. Blots were done on two animals.

G. Body weight and food intake measurements show no differences between WT and ZAK KO mice fed with normal and leucine-deficient diets (n = 3 biological replicates, mean ± SEM).

H. Schematic diagram of findings. Under certain stress conditions such as nutrient-poor environment or Rptor deletion, ribosomes are impaired, leading to the activation of ZAKα and consequente trigger of the SRC-YAP pathway, allowing for the emergence of fetal-like intestinal stem cells. Created with BioRender.com.
